# Theoretical Study of High Harmonic Generation in Monolayer NbSe_2_

**DOI:** 10.1021/acs.jpcc.5c05450

**Published:** 2026-01-19

**Authors:** Daniel A. Rehn, Towfiq Ahmed, Prashant Padmanabhan, Jinkyoung Yoo, Rohit Prasankumar, Jian-Xin Zhu

**Affiliations:** † Computational Physics Division, 5112 Los Alamos National Laboratory, Los Alamos, New Mexico 87545, United States; ‡ National Security Directorate, 6865 Pacific Northwest National Laboratory, Richland, Washington 99354, United States; § Center for Integrated Nanotechnologies, Los Alamos National Laboratory, Los Alamos, New Mexico 87545, United States; ∥ Theoretical Division, Los Alamos National Laboratory, Los Alamos, New Mexico 87545, United States; ⊥ Enterprise Science Fund, Intellectual Ventures, Bellevue, Washington 98005, United States

## Abstract

High harmonic generation (HHG) is a powerful probe of electron dynamics on attosecond to femtosecond time scales and has been successfully used to detect electronic and structural changes in solid-state quantum materials, including transition-metal dichalcogenides (TMDs). Among TMDs, bulk NbSe_2_ exhibits charge density wave (CDW) order below 33 K and becomes superconducting below 7.3 K. Monolayer NbSe_2_ also has superconducting and CDW behavior and is therefore interesting as a material whose different structural and electronic properties could be probed via HHG. Here, we predict the HHG response of the pristine 2H and CDW phases of monolayer NbSe_2_ using real-time time-dependent density functional theory under the application of a simulated laser pulse excitation. We find that due to the lack of inversion symmetry in both monolayer phases, it is possible to excite even harmonics and that the even harmonics appear as the transverse components of the current response under excitations polarized along the zigzag direction of the monolayer, while odd harmonics arise from the longitudinal current response in all excitation directions. This suggests that the even and odd harmonic responses can be controlled via the polarization of the probing field, opening an avenue for potentially useful applications in optoelectronic devices.

## Graphical abstract

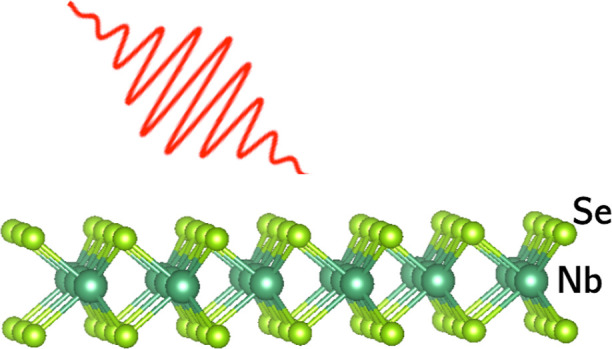

## 1 Introduction

Transition-metal dichalcogenides (TMDs) have attracted much attention recently for their useful and novel physical properties.^1,2^ In bulk form, most TMDs exist as stacked two-dimensional layers where the atoms within each layer exhibit strong covalent bonding, and layers are held together by weaker van der Waals interactions. These bulk structures can be exfoliated down to a single layer (i.e., monolayer), and most TMD monolayers exhibit interesting properties not found in the bulk. Many monolayer TMDs exhibit structural polymorphism, in which the individual layers can exist in multiple structural phases that can be exploited for a variety of applications.^3^ As such, monolayer TMDs are currently being investigated for a wide range of potential applications, such as their use in phase-change memory,^4–6^ optoelectronics,^7^ sensors,^8–10^ straintronic devices,^2,11^ and more.

Several monolayer and bulk TMDs exhibit charge density wave (CDW) order and superconductivity at low temperatures, as seen in NbS_2_,^12–16^ NbSe_2_,^17–36^ TaS_2_,^37–40^ and TaSe_2_,^41–44^ making them potentially useful for controlling these quantum phases with external stimuli, not only in the form of temperature but also with other nonthermal external stimuli such as electrostatic doping^35^ and strain.^36^

In recent years, high harmonic generation (HHG) has been studied in the context of both bulk^45–52^ and monolayer materials^53–62^ with important applications. HHG has been used as a probe to detect the 2H-to-1T′ structural phase change in monolayer MoTe_2_ upon electrostatic doping,^6,63^ topological phase transitions,^64,65^ and to study harmonic orders of up to 13 in monolayer MoS_2_.^53^ In addition, recent experiments have shown the appearance and tunability of noninteger harmonics in the topological insulator Bi_2_Te_3_.^66^ Such studies demonstrate the utility of HHG as a useful attosecond to femtosecond probe of a wide range of materials that can be used in a variety of applications.

Solid-state HHG on quantum materials has become a rapidly intensifying topic of research, though HHG studies on superconductors and CDW materials in particular have been relatively sparse, particularly for monolayer systems, where we are unaware of any experimental studies. Recently, HHG on bulk NbSe_2_ has been performed,^67^ though these experiments were performed at room temperature on the 2H phase and did not study the crossover to the CDW phase. So far, no measurements have been made on monolayer NbSe_2_. HHG on the high-*T*_c_ superconductor YBCO thin films was recently performed and carried out across the superconducting transition temperature.^68^ Additionally, a recent study on films of TiSe_2_ measured HHG across the CDW transition temperature.^69^ In analogy to ongoing questions regarding the influence of topology on HHG,^70^ unambiguous correlations between the presence of superconducting phases and CDW order and the emergence/suppression of harmonics, their relative intensities, their polarization dependencies, and the overall bandwidth (i.e., cutoff) of emission are yet to be identified. Here, analytical and first-principles approaches for the prediction of solid-state HHG profiles are of vital importance if we are to establish the HHG as a versatile and robust all-optical probe of emergent phenomena in quantum materials.

In this work, we study HHG in monolayer NbSe_2_ using real-time time-dependent density functional theory (RT-TDDFT) simulations. By exciting the monolayer with a simulated femtosecond laser pulse, we can predict the current response and HHG spectra under a variety of pulse orientations and strengths as the real-time propagation allows us to probe both the linear and nonlinear electronic response regimes. Doing so, we find that monolayer NbSe_2_ exhibits a strong anisotropy in the strength of the transverse current response when excited along the [100] crystal axis (*x* or zigzag direction, oriented along the **a** lattice constant in Figure 1) and [010] crystal axis (*y* or armchair direction, perpendicular to **a** in Figure 1).^2^ In the case of excitation along the armchair direction, we find that the even modes are largely suppressed for both the longitudinal (current parallel to pulse orientation) and transverse (current perpendicular to pulse orientation) current response. However, for laser pulses oriented along the zigzag direction, we find that the transverse components of the current response are strongly amplified for excitation strengths of 10^11^ and 10^12^ W/cm^2^, well above the linear response regime.

**Figure 1:**
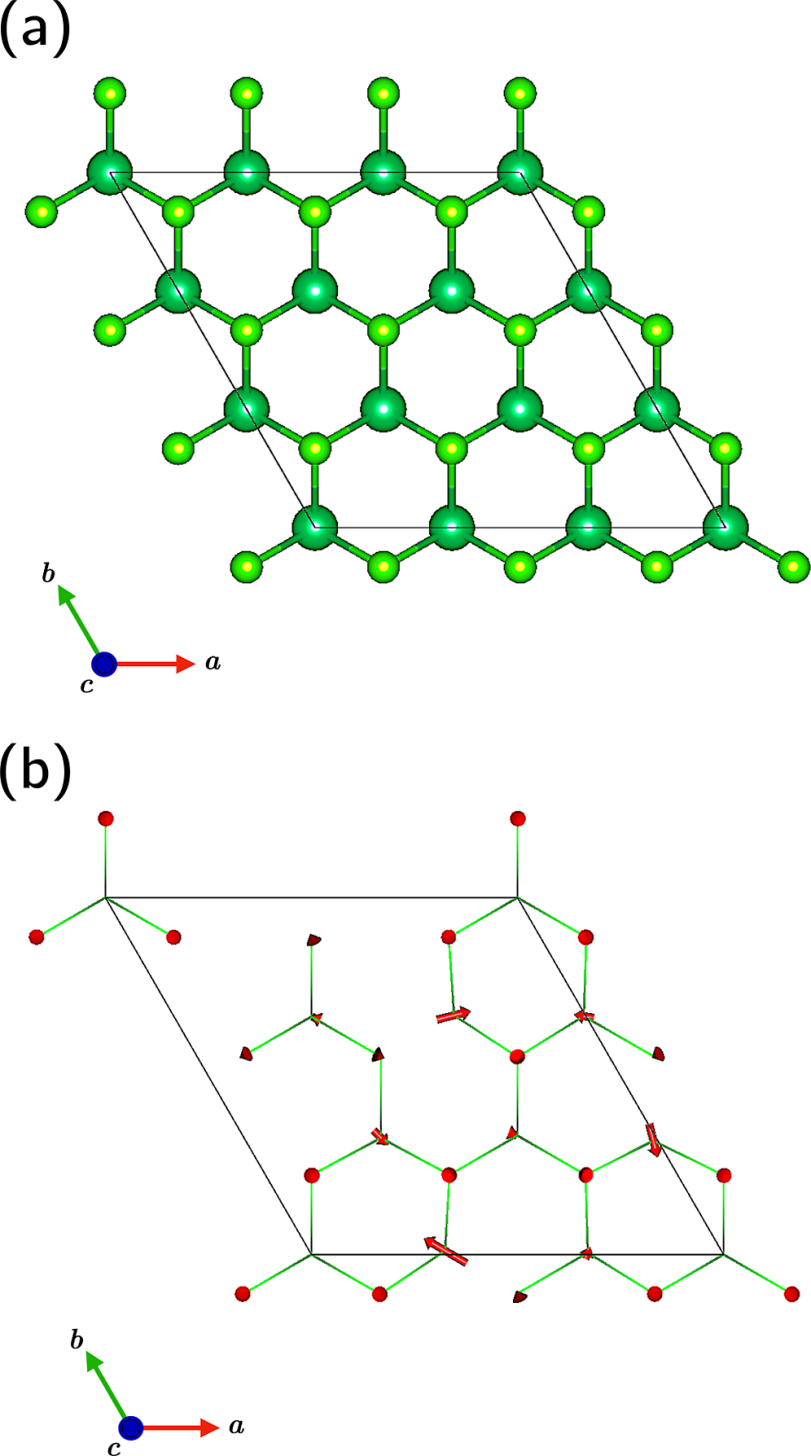
Unit cell of (a) 2H monolayer and (b) a monolayer of the CDW phase, with arrows representing enlarged atomic distortion directions from the 2H monolayer. Nb atoms are dark green, Se atoms are light green. Simulations are performed for both cells shown, each of which consists of 27 atoms.

In the case of excitation along the zigzag direction, the longitudinal HHG spectrum consists primarily of odd-numbered multiples of the excitation frequency (*n*ω_0_, *n* = 1, 3, 5,··· with ω_0_ the laser pulse frequency). The inversion symmetry breaking of the 2H monolayer, as compared to its 2H bulk form, allows in general for even modes (*n*ω_0_, *n* = 2, 4,···) to be present. We find that these even modes appear in the HHG spectra as the transverse components of the current for pulses oriented along the zigzag direction. In this sense, the transverse HHG spectra under excitation along the zigzag direction could be exploited for use in device applications. To summarize, the odd-numbered modes of the HHG response are present in excitations oriented along both the zigzag and armchair directions, but the even mode response occurs only for excitations along the zigzag direction, and in that case, the even modes occur only in the transverse direction, so that the direction-dependence of both the exciting laser pulse and measurement orientation may be exploited.

In addition to the current response, we investigated the effect of CDW distortion on the HHG spectrum of monolayer NbSe_2_. We find that the CDW distortion, determined from prior experiments, does not lead to an appreciable change in the HHG spectrum as compared to the pristine 2H monolayer. This is a result of the small atomic displacements of the CDW phase as compared to the 2H phase that lead to only small changes in the electronic structure of the CDW phase. To understand whether this would remain the case under larger distortions, we perform calculations under enlarged distortion conditions. We find that for these enlarged distortions, noticeable changes arise in the HHG spectra only when the ground-state electronic structure changes appreciably from the 2H phase. This indicates the possibility that for materials exhibiting a larger structural change upon CDW formation than in NbSe_2_, the pristine-to-CDW structural change could lead to distinct changes in the HHG spectra, opening the possibility of probing CDW distortion on femtosecond time scales. We anticipate that the methodology we present will help to better isolate unambiguous signatures of structural symmetry and distortions in regard to CDWs, while also providing a framework to identify candidate systems where such signatures may be significantly enhanced either naturally or through engineering (e.g., in moiré systems^71^). Moreover, while we have focused on the CDW order, we expect that our RT-TDDFT approach can be generalized to a diverse array of materials, providing much needed insight into the connection between HHG and complex intrinsic properties (e.g., topologically nontrivial states).

## 2 Methods

The real-time formulation of time-dependent density functional theory^72^ is carried out in a time-dependent Kohn–Sham (KS) scheme in which the KS wave functions ψ_*i*_(**r**, *t*) are evolved via^73^

1 $$i\hbar \frac{\partial }{\partial t}\psi_{i}\left(r,t\right)={Ĥ}_{KS}\psi_{i}\left(r,t\right)$$

where *i* is the band index and

2 $$\begin{array}{l} {Ĥ}_{KS}=\frac{p^{2}}{2m}{+v}_{ion}\left(r\right)+e^{2}\int dr^{\prime }\frac{n\left(r^{\prime },t\right)}{\left|r-r^{\prime }\right|}\\ +{\;v}_{xc}\left[n\left(r,t\right)\right]+eE\left(t\right)\cdot r. \end{array}$$

Here **p** = −*iℏ*∇, *m* is the electron mass, and *v*_ion_(**r**) is the potential of the ions, assumed here to be static and treated using pseudopotentials as discussed below. The third term is the Hartree Coulomb interaction, the fourth term *v*_xc_[*n*] is the exchange–correlation potential, and the electron density is

3 $$n\left(r,t\right)=\sum_{i}|\psi_{i}\left(r,t\right){|}^{2}$$

**E**(*t*) is the time-dependent electric field. The time-dependent external interaction potential term *e* **E**(*t*)·r breaks the translational symmetry of periodic systems. This can be addressed by adopting the velocity gauge, where a vector field, defined as^74^

4 $$A\left(t\right)=-c\int^{t}E\left(t^{\prime }\right)dt^{\prime }$$

is used to gauge-transform the KS wave functions as^75^

5 $$\psi \left(r,t\right)\to \exp\left[\frac{ie}{\hbar c}A\left(t\right)\cdot r\right]\psi \left(r,t\right)$$

and the velocity gauge TDKS Hamiltonian now takes the form

6 $$\begin{array}{l} {Ĥ}_{KS}^{RT}=\frac{1}{2m}{\left[p+\frac{e}{c}A\left(t\right)\right]}^{2}+v_{ion}\left(r\right)\\ +e^{2}\int dr^{\prime }\frac{n\left(r,t\right)}{\left|r-r^{\prime }\right|}+v_{xc}\left[n\left(r,t\right)\right] \end{array}$$

The coupling to the external field is now incorporated in the kinetic energy term, and consequently the translational symmetry of the Hamiltonian is restored. The time-dependent KS orbitals are now evolved using

7 $$i\hbar \frac{\partial }{\partial t}\psi_{i}\left(r,t\right)={Ĥ}_{KS}^{RT}\psi_{i}\left(r,t\right)$$

In RT-TDDFT, a time evolution propagator is needed to evolve eq 7 in time. Several options for doing so for solids have been discussed in the literature,^76–79^ with a detailed discussion found in ref 76.

By propagating the KS wave functions, the time-dependent current density

8 $$j\left(t\right)=-\frac{i}{2\Omega }\int_{\Omega }dr\sum_{i}\left[\psi_{i}^{*}\left(r,t\right)\nabla \psi_{i}\left(r,t\right)-c.c.\right]$$

can be obtained, whose power spectrum gives the HHG response

9 $$HHG\left(\omega \right)=\omega^{2}{\left|\int_{0}^{T}j\left(t\right)\exp\left(-i\omega t\right)dt\right|}^{2}$$

where Ω is the spatial volume of the unit cell and *T* in the Fourier transformation is the total propagation time for our simulation.

The HHG spectrum is computed from the finite-time Fourier transform of the cell-averaged current, eq 9, over 0 ≤ *t* ≤ *T*, where *T* = *T*_1_ + *T*_2_ with *T*_1_ = 30 fs (pulse duration) and a short post-pulse window *T*_2_. To mitigate spectral leakage inherent to the finite window, we multiply the time trace by a smooth full-window taper

10 $$w\left(t\right)=1-3{\left(t/T\right)}^{2}+2{\left(t/T\right)}^{3},\left(0\leq t\leq T\right)$$

so that *j*(*t*) → *j*_win_(*t*) = *j*(*t*)*w*(*t*). This polynomial window equals 1 at *t* = 0 and vanishes at *t* = *T* with zero slope at both end points. We also verified that the spectra are qualitatively unchanged for total times *T* in the range 30–35 fs (see the Supporting Information).

Also note that all calculations are performed for fixed atomic positions, since the maximum simulation times are on the order of tens of femtoseconds, much faster than the vibrational time scales of the nuclei. In this frozen-ion approximation, thermal motion over the ∼31 fs window primarily leads to inhomogeneous broadening/dephasing and reduced absolute yields, but it does not alter the symmetry-based selection rules and relative trends emphasized here (see the Supporting Information).

The velocity gauge formalism for RT-TDDFT has been implemented in several codes, including TDAP,^80^ RT-SIESTA,^81^ Elk,^82,83^ OCTOPUS,^84^ and SALMON,^85^ among others. We used SALMON version 2.0.2 for all calculations presented in this article. SALMON is a real space code with options to apply periodic boundary conditions, so it is well-suited for studying monolayer NbSe_2_.

All calculations are performed for fixed atomic positions since the maximum simulation times are on the order of tens of femtoseconds, much faster than the vibrational time scales of the nuclei. In addition, all calculations are performed under the adiabatic approximation within TDDFT, which is to say that we neglect the history dependence in the evaluation of the exchange-correlation functional at each time and instead evaluate *v*_xc_[*n*] using instantaneous *n*(**r**, *t*). Within the adiabatic approximation, we employ the local density approximation (LDA)^86^ for all calculations. SALMON makes use of pseudopotentials, and for all calculations, we use Trouiller–Martin type norm-conserving LDA-FHI pseudopotentials^87,88^ for the Nb and Se atoms, where 5 (4d^4^5s^1^) and 6 (4s^2^4p^4^) electrons were treated in the valence, respectively. We verified the accuracy of these pseudopotentials for reproducing the ground-state electronic structure of monolayer NbSe_2_ by comparing the ground-state density of states (DOS) to highly accurate all-electron calculations performed in the Elk code^83^ version 7.0.12 (see Supporting Information Section S3 for details). The density of states we calculate is in good agreement with X-ray photoemission experiments.^89^ Also note that we do not include spin–orbit coupling (SOC) effects in this work as this capability is not available in the code at present. SOC effects were studied recently in the context of the CDW phase.^90^

For all calculations, we used hexagonal unit cells for both the pristine 2H monolayer and CDW monolayer that consist of 9 formula units (f.u.) of NbSe_2_ (27 atoms in total). The pristine monolayer and CDW space groups are both *P*6̅_2_ *m* (#187) within the standard tolerances of symmetry software used.^91^ The monolayer structures are found by isolating a single layer of the bulk 2H phase, space group *P*6_3_/*mmc* (#194), and CDW phase, space group *P*6_3_/*m* (#176). For the CDW phase, the 9 f.u. cell corresponds to the primitive cell, while for the 2H phase, this corresponds to a 3 × 3 × 1 supercell (see Figure 1). In order to isolate the effects of CDW distortion in our calculations, we chose to use the same lattice constants for both the pristine and CDW phases, where we use the CDW phase lattice constants determined by Malliakas et al. (ref 21) measured at 15 K, which is |*a*| = |*b*| = 10.3749 Å. This corresponds to a primitive 2H unit cell size of |*a*| = |*b*| = 3.4583 Å, which is slightly expanded from the 2H lattice constants measured at room temperature, |*a*| = |*b*| = 3.442–3.446 Å, as determined by various sources.^21,92^ This corresponds to an axial strain of the pristine cell of less than 0.5%, not accounting for differences arising from thermal expansion. As NbSe_2_ is metallic, the use of the bulk experimental lattice constants, which will in general not correspond exactly to the equilibrium (zero temperature) lattice constants of the monolayer predicted within the LDA, is not expected to lead to a significant qualitative change in the RT-TDDFT results. This situation would be different for semiconducting and insulating systems, in which small strains can lead to important qualitative changes that could strongly influence the current response, e.g., stemming from changes from a direct to an indirect band gap. Because the monolayer lattice constants are not as well characterized and depend on the choice of substrate and growth or exfoliation method, we chose to use the experimentally measured bulk CDW lattice constants for all monolayer calculations.

All RT-TDDFT calculations require an initial calculation of the ground state as the initial state used to start the real-time propagation with the laser pulse described above. Because SALMON is a real space code and we use periodic boundary conditions, we must ensure that both the ground state and the HHG spectra are converged with respect to the real-space grid size and *k*-mesh. For the ground state, we determined that a grid density *n*_r_ = 2 pts/bohr is necessary to ensure that the wave functions are adequately represented on the grid (see Supporting Information Section S2 for details). Note that accurate ground-state calculations required some adjustments in the mixing parameters of the Broyden scheme^93^ (see Supporting Information Section S1 for details), and the self-consistency cycle was stopped after $\left(\int_{\Omega }\left|n_{i}\left(r\right)-n_{i-1}\left(r\right)\right|d^{3}r\right)/N_{el}<10^{-10}$ , where *n*_*j*_(**r**) is the charge density computed for the *j*th step in the self-consistency cycle and *N*_el_ is the total number of valence states in the cell.

To determine the *k*-mesh size needed for calculations, we first ensured that the ground-state density of states was converged with respect to the *k*-mesh size and then performed convergence studies of the HHG spectra with respect to the *k*-mesh size. We found that for the 9 f.u. cells, a *k*-mesh of size 8 × 8 × 1 is needed to ensure the HHG spectra are well converged (see Supporting Information Section S5 for details). Also note that no symmetry reductions of the *k*-mesh are used in calculations so as not to enforce unwanted symmetries in the time-dependent Kohn–Sham wave functions.

## 3 Results

### 3.1 Laser Pulse Excitation

Using the methodology discussed in the Methods section, we simulate the application of a femtosecond laser pulse for pulses polarized along both the zigzag armchair directions with a pulse shape whose Cartesian component α = *x*, *y* is defined as

11 $$A_{\alpha }\left(t\right)=-c\frac{E_{o}}{\omega_{o}}\cos\left(\omega_{o}t\right)\sin^{2}\left(\frac{\pi t}{\tau }\right)\Theta \left(\tau -t\right)$$

where the electric field can alternatively be defined as

$$\begin{array}{l} E_{\alpha }\left(t\right)=-\frac{1}{c}\frac{\partial A_{\alpha }\left(t\right)}{\partial t}\\ =\frac{E_{o}}{\omega_{o}}\frac{\partial }{\partial t}\left[\cos\left(\omega_{o}t\right)\sin^{2}\left(\frac{\pi t}{\tau }\right)\Theta \left(\tau -t\right)\right] \end{array}$$ 12

In our calculations, we use a pulse width of τ = 30 fs and a photon energy of ω = 0.6 eV ≈ 145 THz. The intensity of the pulse is related to the amplitude by *I* ≈ *cE*_*o*_^2^/8π. This equality only holds for infinite unmodulated wave trains (not pulses), but the approximation can be used to determine a corresponding value for *E*_0_ in eqs 11 and 12, as described in ref 94. The external field *E*_α_(*t*) pulse shapes for four different intensities, 10^9^, 10^10^, 10^11^, and 10^12^ W/cm^2^, are shown in Figure 2a. All figures here are shown using atomic units (a.u.), unless specified otherwise.

**Figure 2:**
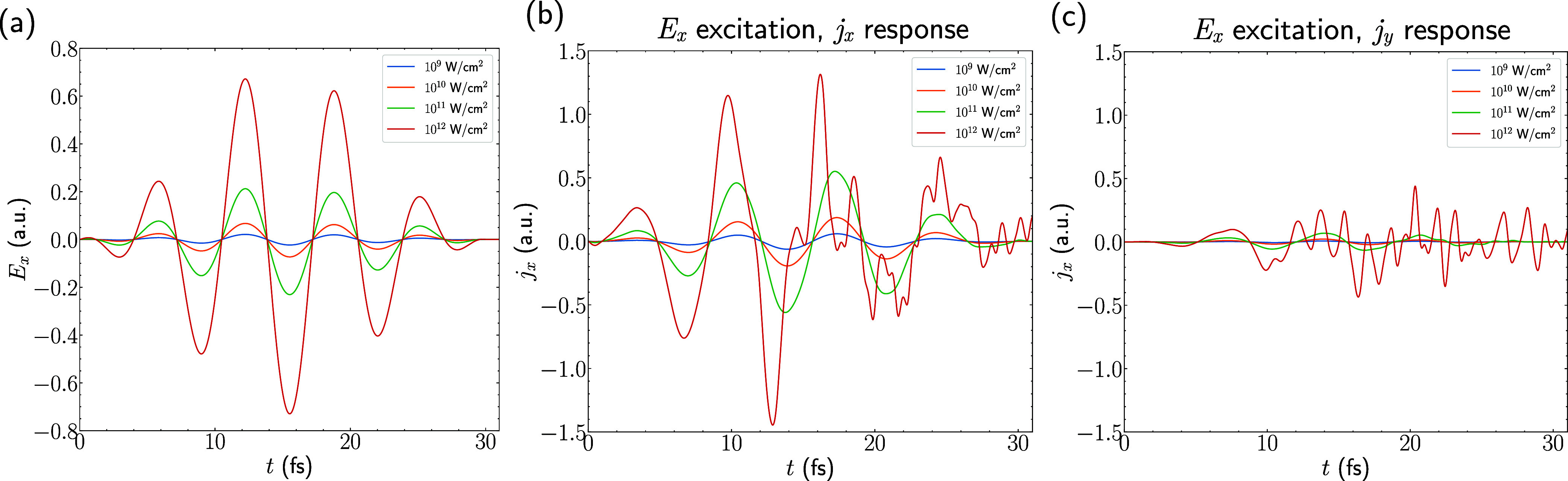
Real-time excitation pulse and current response for excitations along the zigzag (*E*_*x*_) direction, applied to the pristine 2H monolayer. (a) *E*_*x*_(*t*) for different pulse intensities, (b) longitudinal current response *j*_*x*_(*t*) for different pulse intensities, and (c) transverse current response (*j*_*y*_) for different intensities.

Calculations for both CDW and non-CDW structures were performed for a 31 fs time propagation using Δ*t* = 0.08 *ℏ*/*E*_H_ atomic units, or 1.935 attoseconds as the time step. This time step was determined to give the same HHG spectrum as simulations run with Δ*t* = 0.02 *ℏ*/*E*_h_ (see Supporting Information Section 4). For time propagation, we used the enforced time-reversal symmetry propagator.^77^ We also point out that there is a subtle issue associated with how to accurately extract HHG information from RT-TDDFT simulations that use periodic boundary conditions. The issue that arises is that for long enough simulation times, the current can start to propagate through periodic repeats of the cell, which is unphysical and would not be seen experimentally. This issue has been addressed in two different ways. One is to use a multiscale modeling approach in which the microscopic current calculated in the RT-TDDFT approach is used as the input to a macroscopic calculation that solves Maxwell’s equations.^74,95^ The other approach is to run a normal RT-TDDFT simulation that is short enough to avoid the issue of the current interfering with its periodic repeats as a result of the application of periodic boundary conditions and a relatively small cell size.^96^ Note that a related issue is ultrashort dephasing times.^97^ Both approaches have been shown to produce useful qualitative information. Therefore, in this study, we take the second approach and evolve the TDKS equations for only 1 fs after the 30 fs laser pulse. We extensively tested this issue and found that the results do not change much qualitatively for any total simulation time from 30 to 35 fs, as discussed in Supporting Information Section S6. We also point out that experimentally, HHG spectra are usually collected only for the duration of the excitation pulse, in agreement with our computational approach.

### 3.2 Transverse HHG in the 2H Monolayer

With application of the electric field pulse described in the Methods section, we compute the current response throughout the pulse duration (30 fs) and for 1 fs after (31 fs total). As an example, we plot the longitudinal (*j*_*x*_) and transverse (*j*_*y*_) currents under excitation in the *E*_*x*_ direction in Figure 2b,c, respectively. This shows that the transverse components are much smaller in magnitude than the longitudinal current, although they are not negligible for the higher pulse strengths. In addition, we see a clear nonlinear response of the current after the first ∼10 fs for the 10^12^ W/cm^2^ case, and although it is not as obvious for 10^11^ W/cm^2^, the nonlinear response is also present. By nonlinear, we mean that the current response does not respond linearly with the driving field, and higher frequency oscillations in the current appear. The nonlinear transverse currents arise from the nonzero Berry curvature of bands.^98^

In Figure 3, we plot the HHG spectra (calculated via eq 9) for the *E*_*x*_ (panel a) and *E*_*y*_ (panel b) excitations of the pristine 2H monolayer. The blue, orange, green, and red lines correspond to pulse intensities of 10^*n*^ W/cm^2^, *n* = 9, 10, 11, and 12, respectively. The solid lines are the *j*_*x*_ current while the dashed lines are the *j*_*y*_ current. All spectra are obtained from the finite-time Fourier transform in eq 9 over the total propagation time. As is typical for finite windows, the transformation introduces a small, smooth background and ripples (spectral leakage) that are deterministic rather than stochastic noise. We confirmed that the spectra are qualitatively unchanged for total times between 30 and 35 fs (see the Supporting Information), which is also consistent with experiments that predominantly collect HHG during the excitation pulse. To reduce leakage for display only, we apply a smooth end-taper to *j*(*t*) (documented in the Methods section). Because higher orders are intrinsically much weaker than the fundamental, a logarithmic intensity axis is used to display multiple harmonics across the large dynamic range.

**Figure 3:**
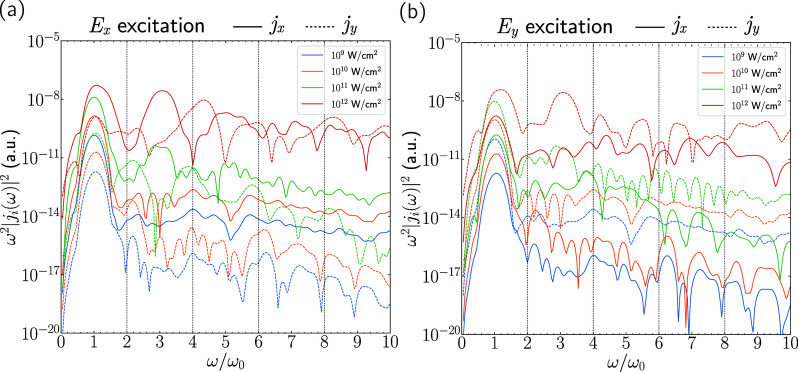
HHG spectra for different excitation strengths in the 2H monolayer. (a) *E*_*x*_ excitation with *j*_*x*_ (solid) and *j*_*y*_ (dashed) response. (b) *E*_*y*_ excitation with *j*_*x*_ (solid) response and *j*_*y*_ (dashed) response.

In Figure 3a, the *j*_*x*_ current is the dominant contribution to the total current, being the longitudinal current response, whereas *j*_*y*_ is the dominant contribution for the *E*_*y*_ excitation, as in that case, it is the longitudinal current. In addition, we see that in the 10^9^ and 10^10^ W/cm^2^ cases, the only prominent response is at the pulse frequency ω = ω_0_. This is a clear indication that these pulse intensities are not strong enough to induce a nonlinear current response that is strong enough to cause the observation of a clear HHG signal. In contrast, we see that for pulse intensities of 10^11^ and 10^12^ W/cm^2^, higher harmonics appear in the longitudinal current response for both *E*_*x*_ excitation (*j*_*x*_ response) and *E*_*y*_ excitation (*j*_*y*_ response). This indicates that these pulse intensities are strong enough to observe HHG.

In addition, we see that for the zigzag (*E*_*x*_) excitation direction, the 10^11^ and 10^12^ W/cm^2^ intensities give rise to a large *j*_*y*_ contribution, i.e., the transverse current response. In fact, for 10^11^ W/cm^2^, the second harmonic is nearly as strong as the fifth harmonic in *j*_*x*_, and in the 10^12^ W/cm^2^ case, the fourth harmonic is nearly as strong as the third harmonic in *j*_*x*_. This switch in even harmonic intensity, with the second harmonic being most prominent at 10^11^ W/cm^2^ and the fourth harmonic being the most prominent at 10^12^ W/cm^2^, is something that could be tested and verified in experiments as the intensities fall well within the experimentally accessible range. Excitation in the armchair (*E*_*y*_) direction does not lead to an appreciable transverse (*j*_*x*_) response in any of the cases. For monolayers with space group *P*6̅_2_ *m*, no inversion symmetry is present, so it is possible to observe even harmonics in the HHG spectrum. This is in contrast to the bulk 2H phase, where an inversion center is present in the van der Waals gap between the NbSe_2_ layers.

For *E*_*x*_ (zigzag) excitation, the Hamiltonian retains vertical mirror σ_*y*_. Because *j*_*y*_ is odd and *E*_*x*_ is even under σ_*y*_, the transverse current is constrained to be an even function of the driving field, *j*_*y*_(*E*_*x*_) = χ_*yxx*_^(2)^ *E*_*x*_^2^ + χ_*yxxxx*_^(4)^ *E*_*x*_^4^ +···, which forbids odd-order contributions in *j*_*y*_ and yields even harmonics (2ω_0_, 4ω_0_,···) in the transverse HHG spectrum. This is consistent with our finding that even harmonics appear as transverse components under zigzag excitation in the inversion-broken monolayer and with the Berry curvature origin of the transverse nonlinear response.

For the *E*_*y*_ (armchair) excitation, even-order transverse terms are symmetry-allowed, *j*_*x*_(*E*_*y*_) = χ_*xyy*_^(2)^ *E*_*y*_^2^ + χ_*xyyyy*_^(4)^ *E*_*y*_^4^ +···. In our calculations, these components are small, and the even harmonics in Figure 3b are present but weak (a clear 2ω_0_ at 10^11^ W/cm^2^ and both 2ω_0_ and 4ω_0_ at 10^12^ W/cm^2^). The suppression arises from near-cancellation of Brillouin-zone contributions tied to the distribution of band velocities and Berry curvature for this orientation, whereas the complementary tensor for *E*_*x*_ (χ_*yxx*_^(2)^) is sizable and yields the strong transverse even harmonics in Figure 3a. Symmetry dictates allowed/forbidden responses; when allowed, the magnitude is set by the electronic structure and must be computed.

The findings above indicate some potential optoelectronic device applications that we now discuss. Because even harmonics are only found for propagation along the [010] (or another symmetry-equivalent) direction and because these even harmonics are only substantially excited for excitation pulses oriented along the [100] direction, it may be possible to use the even harmonic response as a femtosecond switch to pass information along. For example, given a large enough single-crystal monolayer, it would be possible to apply laser pulses oriented along different directions and simultaneously collect the response. Similar to bits that are either 0 or 1, the appearance of an even harmonic within some femtosecond or shorter window could represent a 1, and the nonappearance could represent a 0. If the pulse intensity was also used as an input variable, it could be possible to go beyond binary channels and pass information along 0 (no even harmonic), 1 (second harmonic), 2 (fourth harmonic), etc. By constructing rules for how one type of response (appearance of a particular harmonic) triggers subsequent excitations, a complex network of rules could be constructed to filter information on femtosecond time scales.

### 3.3 HHG in the CDW Monolayer

Having explained the prediction of the transverse HHG response for even harmonics in the pristine 2H monolayer, we now turn to the question of how the HHG response of the CDW phase compares with that of the 2H monolayer. Using the same computational approach, we make comparisons of only the longitudinal current response in Figure 4 (the transverse current responses are shown in the Supporting Information Section S7). We include only the longitudinal current in order to more easily see the comparisons and reduce the clutter in the figure. Clearly, the HHG spectra look extremely similar for both the pristine (dashed lines) and CDW (solid lines) phases. Although we do see some modest differences, particularly for the 10^12^ W/cm^2^ case in panel (b), these differences are likely not prominent enough to be useful for device applications. These findings are also true for the transverse HHG responses shown in Supporting Information Section S7, where again there are some noticeable differences in the 10^12^ W/cm^2^ case, although they may not be large enough to clearly differentiate phases experimentally.

**Figure 4:**
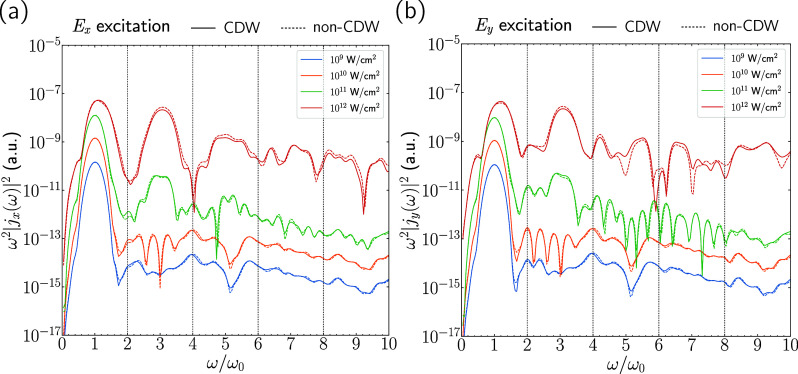
Comparison of the longitudinal current HHG spectra for the CDW (solid lines) and 2H (dashed lines) cells at different pulse intensities. (a) *E*_*x*_ excitation with *j*_*x*_ response. (b) *E*_*y*_ excitation with *j*_*y*_ response. Only the longitudinal components are included in order to reduce clutter; the corresponding transverse components can be found in Supporting Information Section S7.

One interesting question that arises is why the CDW distortion does not lead to a significant change in the HHG response. We point out that since the CDW phase also lacks inversion symmetry, even harmonics may be present. For NbSe_2_, the atomic displacements in moving from the pristine to CDW phase are quite small and comparisons of the ground-state electronic density of states (DOS) show that the distortions are not enough to significantly change the DOS. Although it is not *a priori* clear that close agreement of the ground-state DOS for the two phases is sufficient to say that the HHG spectra will be close, we find that this is borne out in our numerical simulations. However, it will be important to investigate this experimentally as well.

Although we do not find significant differences in the HHG spectra for the CDW and pristine phases of NbSe_2_, the case may be different for other materials with larger CDW structural changes. Therefore, one interesting question is whether an enlarged CDW distortion of the CDW phase of NbSe_2_ could lead to differences in the HHG spectra, which could be made possible through, for example, twist engineering of stacked vdW systems. To explore this, we employ the following technique to generate CDW structures with enlarged distortions. Denoting the positions of the atoms in the pristine 9 f.u. 2H cell as $r_{i}^{2H}\left(i=1,2,\cdot \cdot \cdot 27\right)$ and the positions of atoms in the 9 f.u. CDW cell as **r**_*i*_^CDW^ (*i* = 1, 2,···, 27), we define a new set of atomic positions

13 $$r_{i}^{\alpha }=r_{i}^{CDW}+\alpha \left(r_{i}^{CDW}-r_{i}^{2H}\right)$$

This definition allows us to use different values of α to enhance the structural distortion, with α = 0 corresponding to the experimental CDW structure. In Figure 5, we present comparisons of the longitudinal HHG spectra for *E*_*x*_ and *E*_*y*_ excitations for α = 0, 2, 4 in panels (b) and (c), respectively. For the case of α = 4, the distortion is large enough to start to see noticeable qualitative changes in the HHG spectra. This is also true for the transverse HHG spectra shown in Supporting Information Section S8, where we see perhaps even larger qualitative changes to the HHG spectra.

**Figure 5:**
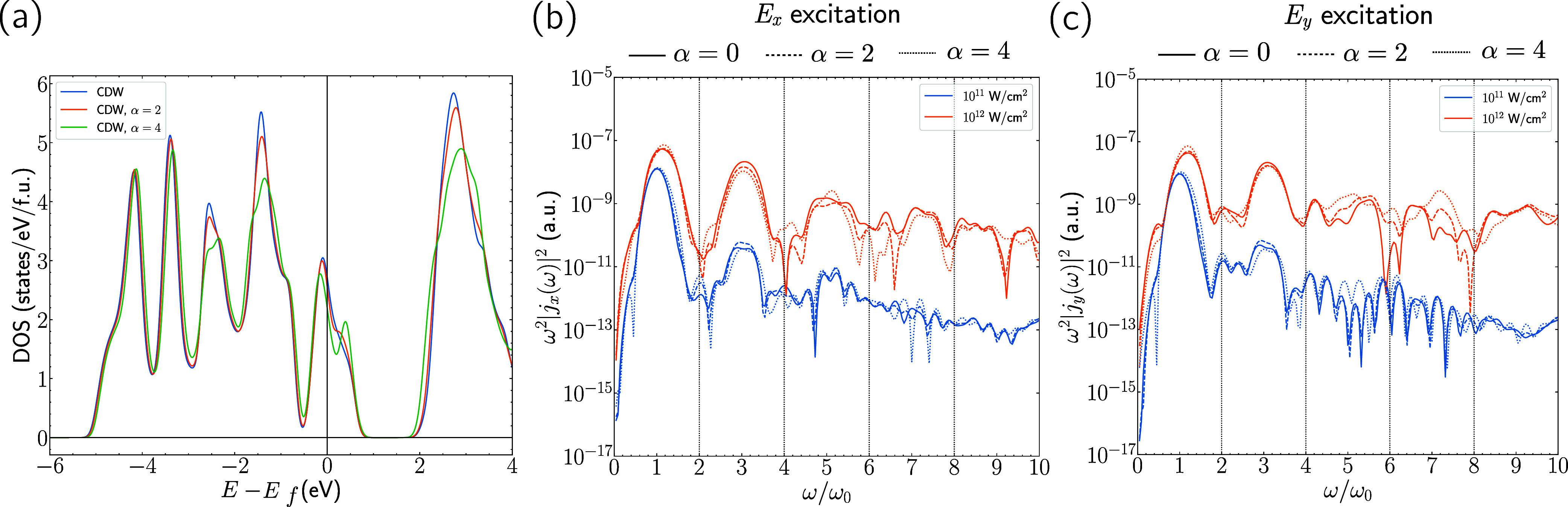
(a) Ground-state DOS for the CDW (α = 0) and enlarged distortion (α = 2, 4) structures. (b) Longitudinal HHG spectra for *E*_*x*_ excitation and *j*_*x*_ response and (c) Longitudinal HHG response for *E*_*y*_ excitation and *j*_*y*_ response, for 10^11^ (blue) and 10^12^ (orange) W/cm^2^ pulse intensities for the three values of α.

The case of α = 4 also produces a significant change in the ground-state DOS as compared to the α = 0 case, as seen in Figure 5a. Therefore, in these calculations, one indicator for whether we might expect to see qualitative changes in the HHG spectra of CDW and non-CDW phases of other materials is if their ground-state DOS are significantly different. Of course, CDW distortions that lead to changes in the space group, particularly in cases where an inversion symmetry is broken, could also be expected to lead to large qualitative differences in the HHG spectra. In this case, both α = 2, 4 have space group *P*6̅ (#174), which is a lower symmetry than the CDW phase and where inversion symmetry is again absent.

The above results show that in principle, larger CDW distortions can lead to distinct HHG spectra that could be detected experimentally and potentially exploited for device applications. Although these effects are relatively small for monolayer NbSe_2_, other materials that exhibit large structural distortions could be interesting to explore both in simulations and experiments.

## 4 Discussion

We have presented results from a computational study of laser pulse excitations of monolayer NbSe_2_ in its pristine 2H and CDW phases. Different pulse orientations and strengths lead to the following predictions: (1) the longitudinal current response for excitations along both the zigzag and armchair directions of NbSe_2_ leads to an HHG signature that is dominated by odd harmonics for excitation strengths of ∼10^11^ W/cm^2^ and higher; (2) the transverse HHG response is extremely pronounced in case of excitations along the zigzag direction and appear predominantly as even harmonics, whereas excitations along the armchair direction do not lead to such a prominent appearance of even harmonics in neither the longitudinal nor transverse HHG components; (3) the second harmonic is the strongest even mode at 10^11^ W/cm^2^, while the fourth harmonic becomes the strongest even mode at 10^12^ W/cm^2^, a finding that could be tested experimentally; (4) the HHG response of the experimentally measured CDW phase does not reveal any significant changes from the pristine 2H phase, due to the small atomic displacements, however (5) enlarging the CDW distortion leads to significant qualitative changes in the HHG spectra, indicating that other materials with larger CDW structural distortions than those of NbSe_2_ may lead to distinct differences in HHG spectra.

While HHG measurements on superconducting and CDW materials have not been explored extensively in experiments so far, we have shown a promising path forward for connecting theory and experiment through the use of real-time TDDFT simulations. Future studies combining both experiments and theoretical calculations will help to establish a deeper connection between highly nonlinear electron dynamics and underlying structural symmetries, the key to correlating HHG emission to underlying order in materials.

Our results indicate that HHG is most sensitive to CDW order when the distortion produces a substantial change in low-energy electronic structure or symmetry. For monolayer NbSe_2_, the experimentally determined CDW distortion yields spectra remarkably similar to those of 2H, in line with the small DOS changes; systematic scaling of the distortion shows that qualitative HHG differences emerge only once the ground-state DOS is appreciably modified (Figure 5). By contrast, materials with stronger CDW distortions provide promising targets. As one example, twisted 1T-TiTe_2_/1T-TiSe_2_ heterobilayers^71^ exhibit moiré-enhanced CDW domains that persist to room temperature and display clear local DOS changes together with larger local strain in the CDW domains than in normal domains, consistent with stronger structural modulation.

The findings summarized here indicate some potential ways to exploit them for useful optoelectronic device applications. In particular, since the even harmonics appear strongly only as the transverse part of the current response for excitations along the zigzag direction, it may be possible to use the detection of even harmonics as a binary, ternary, or higher order switch to pass along information. The potential benefit for optoelectronic applications is that devices could be made atomically thin and operate on femtosecond or faster time scales. Experimental investigation of the predictions presented here would shed light on the feasibility of using NbSe_2_ or other materials for this, and other, device applications.

## Supplementary Material



## Data Availability

All data supporting the findings of this study are available from the corresponding author upon a reasonable request.

## Notes

Convergence of Δ*q* with respect to scf iteration for different *k*-mesh sizes; ground-state DOS for different real space grid density *n*_r_; convergence study of the DOS with respect to *k*-mesh size within SALMON; HHG spectra for different timesteps Δ*t* for a simulation; convergence study of the HHG spectra of the 2H monolayer for different *k*-mesh sizes; convergence of the HHG spectra with respect to post-pulse time; 2H and CDW monolayer comparisons of the transverse HHG spectra; and comparisons of the transverse HHG spectra for exaggerated CDW distortions (PDF)
