# Supplementary material for: Theoretical Study of High Harmonic Generation in Monolayer NbSe2
Source: J Phys Chem C Nanomater Interfaces. 2026 Jan 19;130(4):1551–61. doi: 10.1021/acs.jpcc.5c05450 (PMC12862804; doi:10.1021/acs.jpcc.5c05450)
Supplement: Supplementary file 1 [file jp5c05450_si_001.pdf]

Supplemental Material for Theoretical study of high harmonic generation in monolayer $NbSe_2$

Daniel A. Rehn*[1], Towfiq Ahmed[2], Prashant Padmanabhan[3], Jinkyoung Yoo[3], Rohit Prasankumar[3], and Jian-Xin Zhu†[3,4]

[1]Computational Physics Division, Los Alamos National Laboratory, Los Alamos, New Mexico 87545, USA
[2]National Security Directorate, Pacific Northwest National Laboratory, Richland, Washington 99354, USA
[3]Center for Integrated Nanotechnologies, Los Alamos National Laboratory, Los Alamos, New Mexico 87545, USA
[4]Theoretical Division, Los Alamos National Laboratory, Los Alamos, New Mexico 87545, USA

1 Ground state convergence of the charge density

In this section, we assess the convergence of the ground state charge density, which is measured by the value

$$\Delta q = \frac{1}{N_{\rm el}} \int_{\Omega} |n_j(\mathbf{r}) - n_{j-1}(\mathbf{r})| d^3 r \quad (1)$$

where $N_{\rm el}$ is the total number of electrons, Ω the cell volume, and $n_i(\mathbf{r})$ is the charge density at iteration i of the self-consistent field (scf) cycle. In Fig. S1a we show Δq for different k-mesh sizes of the 9 f.u. (27 atom) 2H monolayer cell at the default Broyden mixing value of $\alpha = 0.75$. Notably, even after 300 scf iterations, Δq does not fall below 10^{-6}. However, when using $\alpha = 0.1$ (purple line) we can achieve $\Delta q < 10^{-10}$. In Fig. S1b we show the scf convergence for different k-mesh sizes for the primitive (3 atom) 2H cell using $\alpha = 0.1$ only. The ground state converges well for this choice of α, regardless of k-mesh size. Therefore $\alpha = 0.1$ is used for all calculations in the main text.

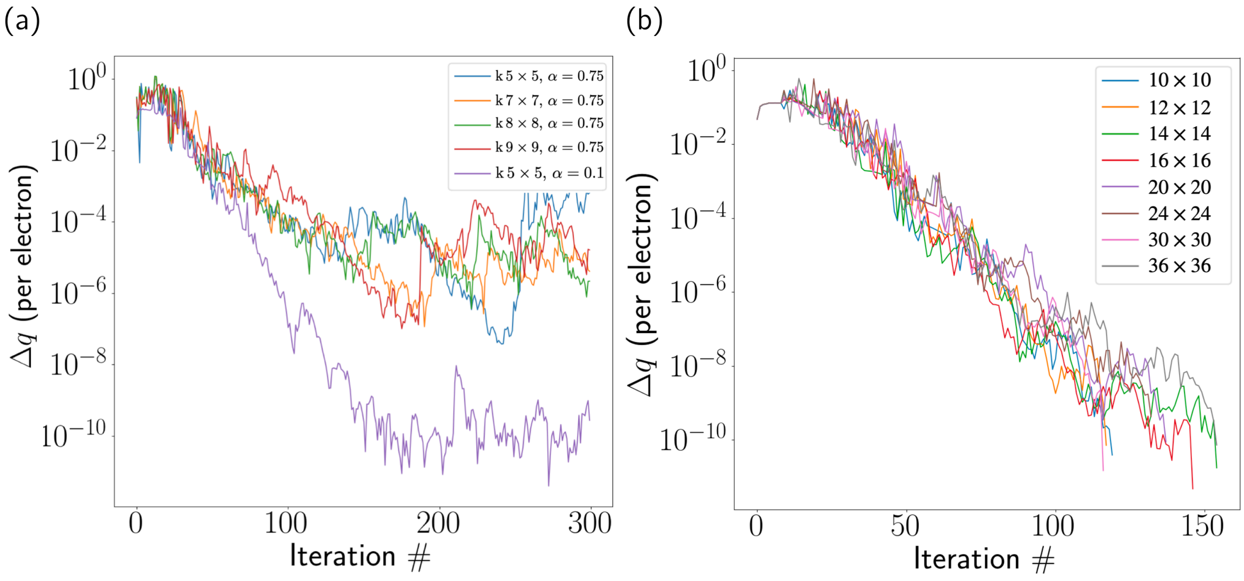

Figure S1: (a) Convergence of Δq with respect to scf iteration for different k-mesh sizes in the 9 f.u. cell with the default Broyden mixing parameter $\alpha = 0.75$ for all lines except the purple (choice of $\alpha = 0.1$, indicating the importance of reducing α for convergence. (b) Same as a, but for the primitive 2H cell only (3 atoms) and for only $\alpha = 0.1$, indicating that this value of α works regardless of k-mesh size.

*rehnd@lanl.gov
†jxzhu@lanl.gov

2 Ground state DOS convergence with real-space grid size

Here we highlight the importance of choosing a high enough real space grid density for simulations. The ground state DOS is plotted in Fig. S2 for different values of n_r, the number of grid points per bohr along each lattice direction. We find from this that a value of $n_r = 2$ is sufficient to ensure a converged DOS, so we use this value for all calculations presented in the main text. All calculations in Fig. S2 are done for the 9 f.u. cell using an $8 \times 8 \times 1$ k-mesh.

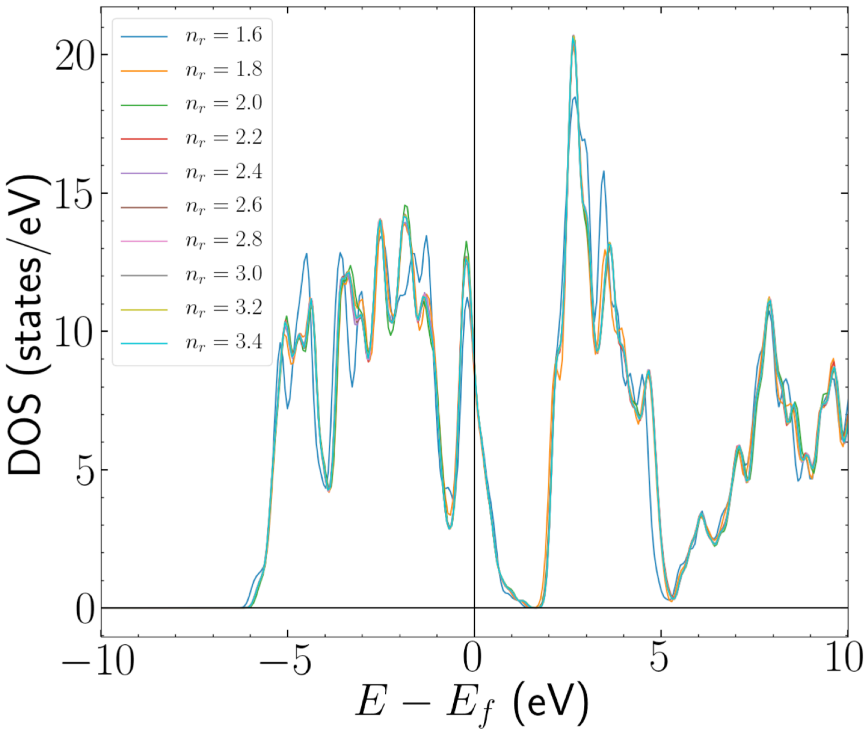

Figure S2: Ground state DOS for different real space grid density n_r (see Sec. 2 discussion).

3 Ground state DOS convergence with k-mesh

In this section, we show two important convergence studies. The first question is whether the density of states (DOS) calculated in SALMON (a real space code) matches other codes. In Fig. S3a we compare the SALMON DOS with the all-electron (FP-LAPW) code Elk, using the same cell size and k-point grid for the 9 f.u. supercell ($8 \times 8 \times 1$) and the LDA in both codes. The two codes agree very well in all aspects. The small discrepancies are due to slight differences in smearing methods. Differences above 5 eV are due to different numbers of bands included in each calculation.

In Fig. S3b we show the DOS computed within SALMON for different k mesh sizes. This shows that for k-mesh sizes above $5 \times 5 \times 1$, the DOS does not change significantly. Note that all lines except the purple line use the default Broyden mixing parameter of $\alpha = 0.75$, with the purple line using $\alpha = 0.1$. As discussed in Sec. 1, it is important to use $\alpha = 0.1$ as the mixing parameter in order to obtain a highly accurate ground state charge density. However, even the cases of $\alpha = 0.75$ shown in Fig. S3b do not show and serious qualitative flaws in the DOS, regardless of k-mesh size. Nonetheless, for all calculations presented in the main text, we use an $8 \times 8 \times 1$ k-mesh and converge the ground state using $\alpha = 0.1$.

Note that all SALMON results in Fig. S3 use a real space grid size of $n_r = 2$ pts/bohr along each of the unit cell axes, i.e., the real space grid points $\Delta r = 0.5$ bohr apart. This was found to be sufficient, as shown in Sec. 2.

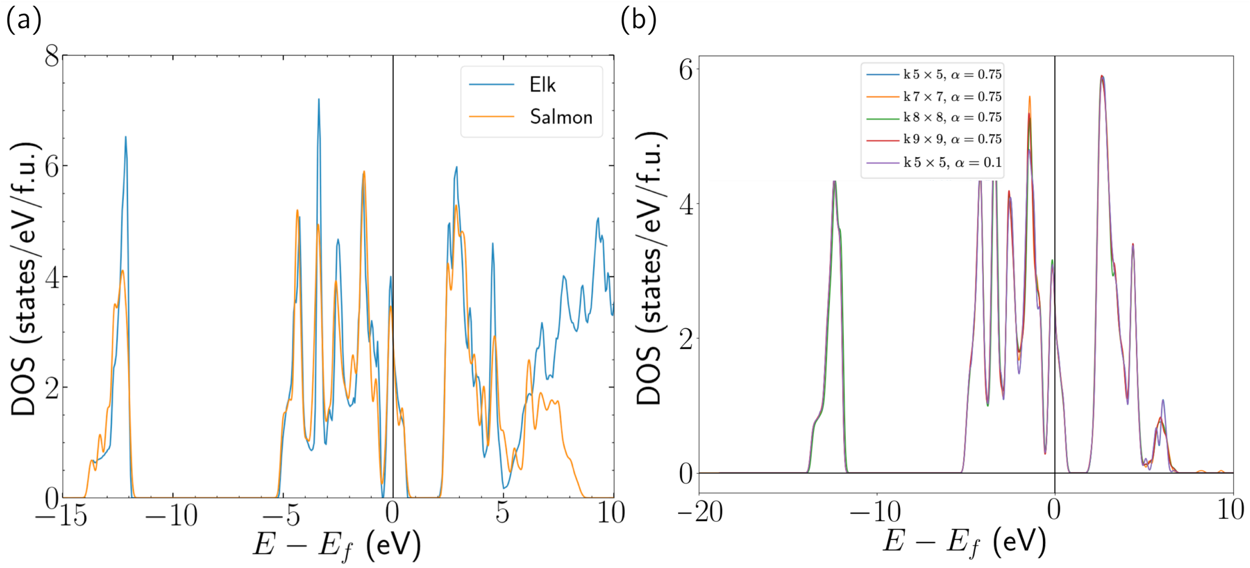

Figure S3: (a) Density of states of the 2H monolayer as computed in Elk (blue) and SALMON (orange) using an 8×8 k-mesh as used in the main text, showing both give very similar results. (b) Convergence study of the DOS with respect to k-mesh size within SALMON. The DOS is less sensitive to the choice of k-mesh than the HHG spectrum (see Sec. 5).

4 Convergence of HHG spectra with timestep

We show in Fig. S4 the HHG spectra calculated with different timesteps Δt (shown in atomic units $\hbar/E_h$). The lines in the figure lie directly on top of each other, indicating that a timestep of 0.08 a.u. $\approx$ 1.9 attoseconds is sufficient.

We also point out that in Fig. S4 the HHG response looks qualitatively different from the results in the main text. This is because the total simulation time T used to calculate the Fourier transform in this figure is $T = T_1 + T_2 = 100$ fs ($T_1 = 30$ fs pulse + $T_2 = 70$ fs after the pulse is turned off). Due to the 70 fs window after the pulse, the current interacts with its periodic repeats, resulting in sharper features in the HHG spectrum. This is unphysical and can be alleviated by running for shorter T_2, as discussed in Sec. 6.

The reason for using $T_2 = 70$ fs for this convergence study is that it allows for a long total simulation time to ensure that any propagation of numerical errors coming from a too-large Δt have a chance to present. However, we do not find any indication of this for any timesteps shown in Fig. S4. We therefore use $\Delta t = 0.08$ atomic units for all simulations in the main text.

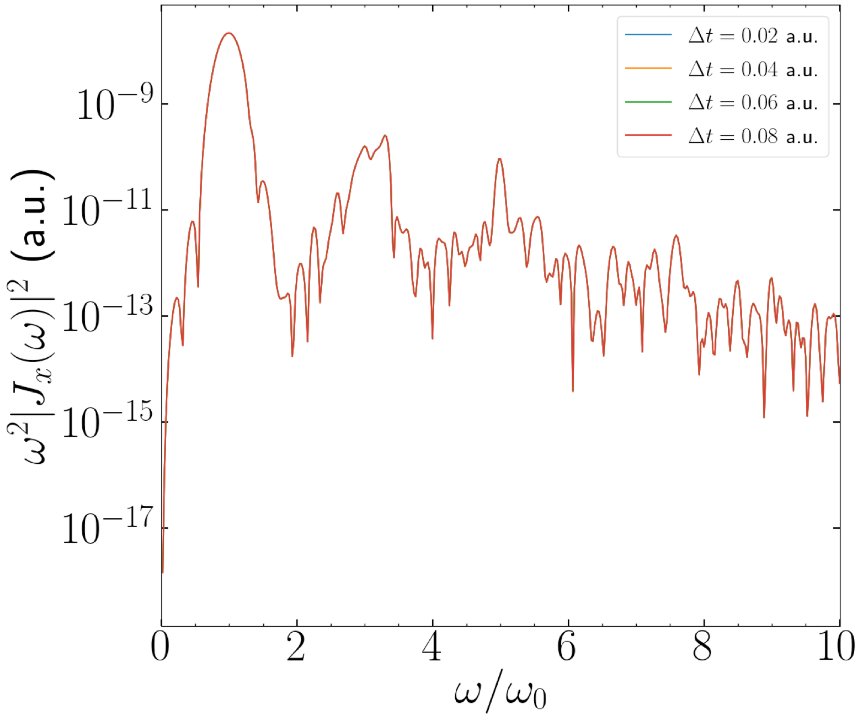

Figure S4: HHG spectra for different timesteps Δt for a simulation with $T_1 = 30$, $T_2 = 70$, $T = 100$ fs to ensure sufficient simulation time to assess integration stability and propagation of numerical errors. This is for E_x excitation of the monolayer with j_x response for 10^{11} W/cm^2. The curves are all overlapping, so $\Delta t = 0.08$ atomic units is sufficiently small.

5 Convergence of HHG spectra with k-mesh

Another question that arises is how the k-point grid influences the calculated HHG spectra. In Fig. S5 we show the HHG spectra of the 2H monolayer for j_x response under E_x excitation at 10^{11} W/cm^2 for a range of k-mesh sizes. We see that the 8×8 mesh is sufficiently close to the higher density grids and therefore use that size for all studies in the main text. Note all simulations use $T_2 = 1$ fs, for $T = 31$ fs.

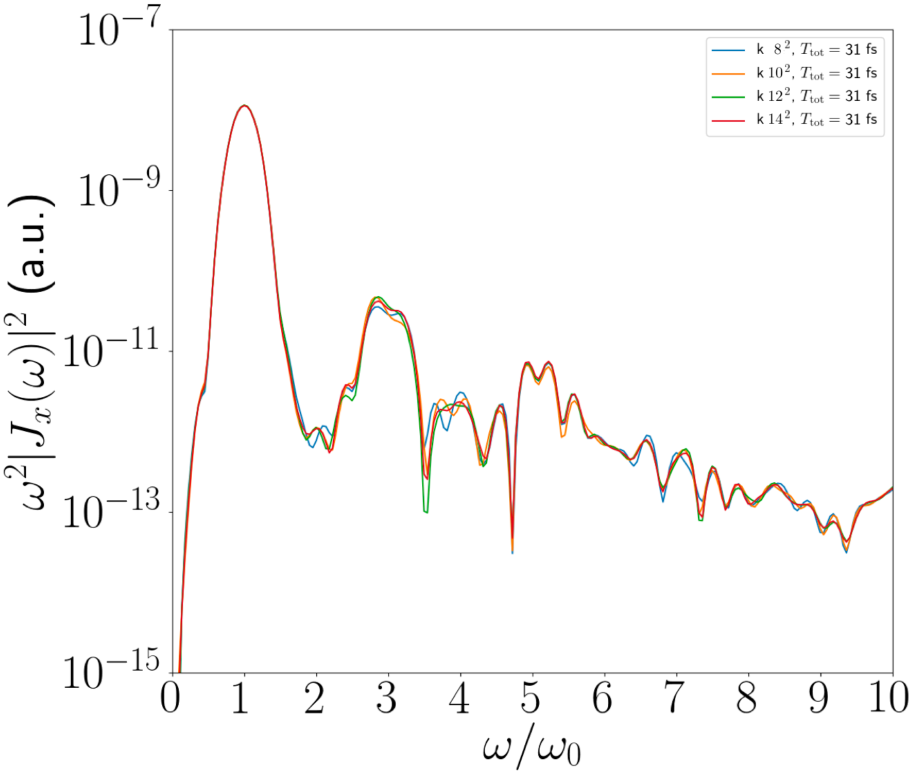

Figure S5: Convergence study of the HHG spectra of the 2H monolayer for different k-mesh sizes. Very small differences can be seen, but a k-mesh size of 8^3 is sufficient to ensure correct qualitative features.

6 Convergence of HHG spectra with T_2

In this section we address the issue of how long to run the simulation after the pulse duration. In fact, it is not strictly necessary to run for longer than the pulse duration. As seen in Fig. 2 of the main text, the nonlinear current response starts during the pulse for large enough pulse strengths. In addition, experimental HHG measurements typically only last for the duration of the pulse. For RT-TDDFT simulations that employ periodic boundary conditions, the issue that arises is that for long enough simulation times the current propagates through the cell and interacts with itself through its periodic images. This issue can be addressed using a multiscale modeling approach in which the microscopic current is used as an input to a macroscopic solution of Maxwell's equations. Alternatively, we can end the simulation before the current has a chance to interact with its periodic images.

In Fig. S6 we show the effect of T_2 (see Sec. 4 for the definition) on the calculated HHG spectra, again for E_x excitation, j_x response for 10^{11} W/cm^2. As T_2 is lowered, some of the sharp features start to disappear and we see a general trend towards convergence. We therefore chose $T_2 = 1$ fs for all simulations presented in the main text.

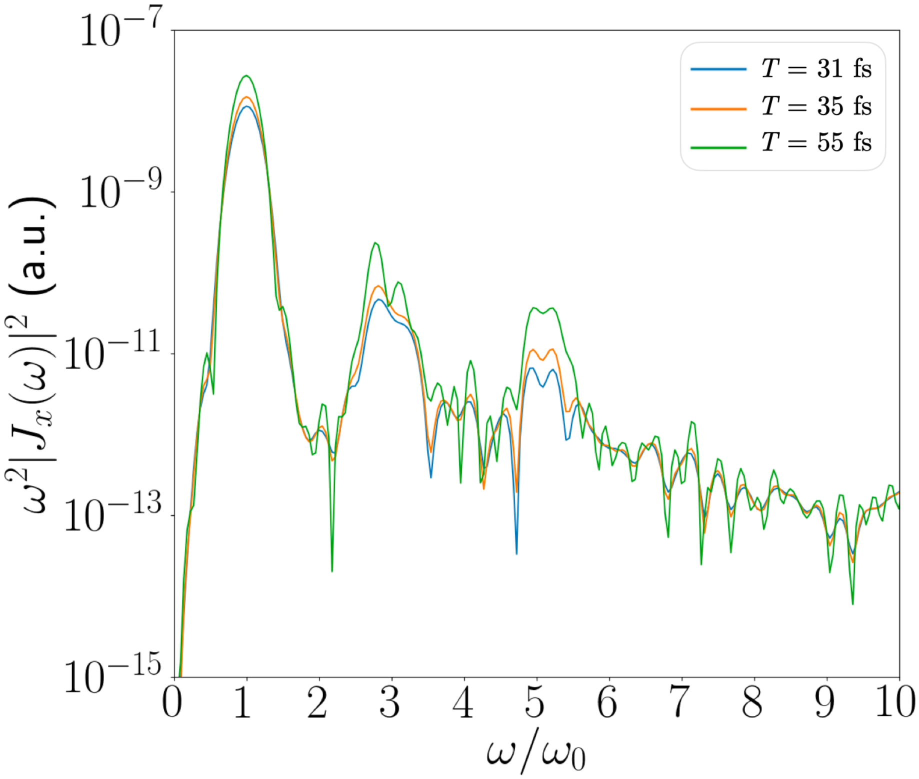

Figure S6: Convergence study of the HHG spectra of the 2H monolayer for different k-mesh sizes. Very small differences can be seen, but a k-mesh size of 8^3 is sufficient to ensure correct qualitative features.

7 2H and CDW transverse HHG comparison

In Fig. S7 we show the transverse HHG spectra comparisons of the CDW and 2H cells. Similar to the results presented in Fig. 4 of the main text, we see overall qualitative similarity, with only small differences.

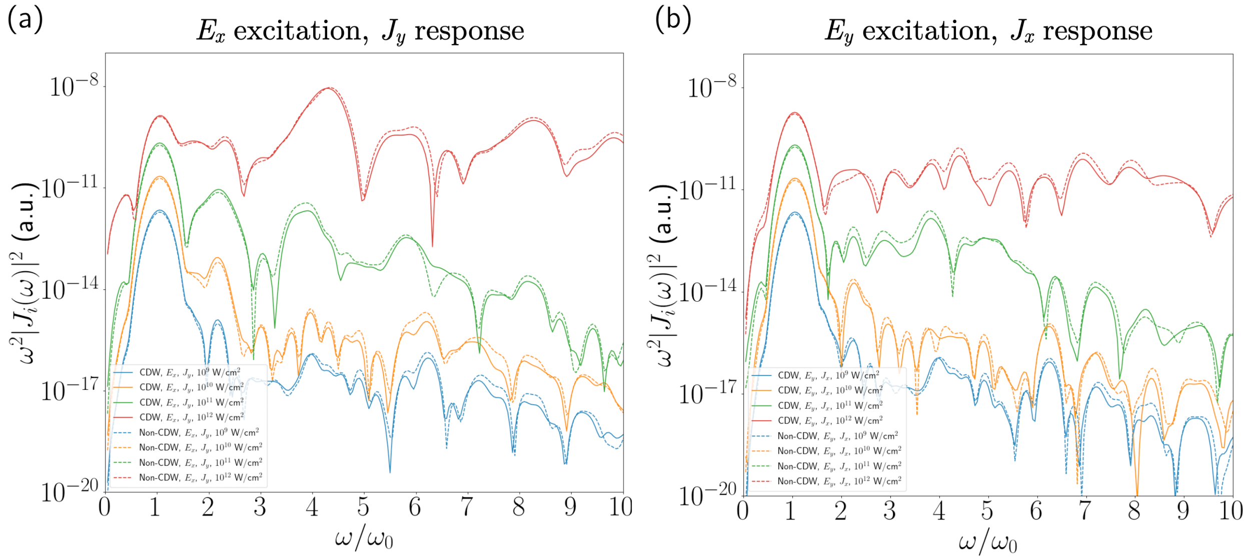

Figure S7: 2H and CDW monolayer comparisons of the transverse HHG spectra. (a) shows the j_y response for E_x excitation and (b) shows the j_x response for E_x excitation.

8 Transverse HHG comparison for enlarged CDW distortion

In Fig. S8 we show the transverse HHG spectra comparisons of the CDW cells with exaggerated distortion. Similar to the results presented in Fig. 5 of the main text, we see larger changes in the HHG spectra as the distortion increases, indicating that other CDW materials with larger structural distortion than in $NbSe_2$ could show unique HHG signatures upon CDW formation.

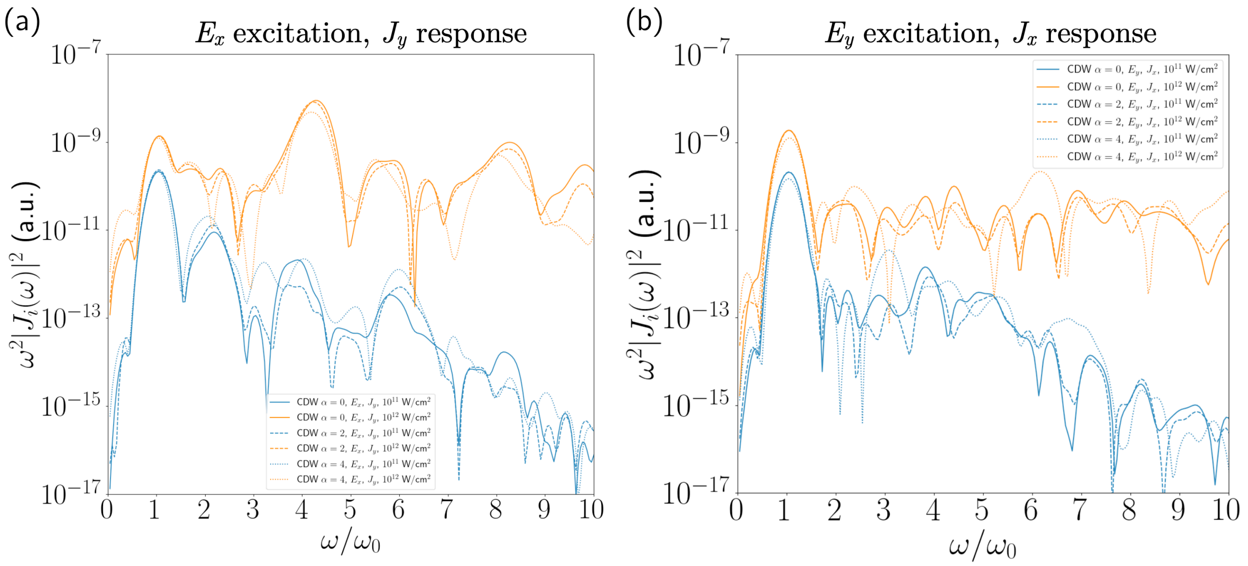

Figure S8: Comparisons of the transverse HHG spectra for exaggerated CDW distortions. (a) shows the j_y response for E_x excitation and (b) shows the j_x response for E_x excitation.
